# Inbred lab mice are not isogenic: genetic variation within inbred strains used to infer the mutation rate per nucleotide site

**DOI:** 10.1038/s41437-020-00361-1

**Published:** 2020-08-31

**Authors:** Jobran Chebib, Benjamin C. Jackson, Eugenio López-Cortegano, Diethard Tautz, Peter D. Keightley

**Affiliations:** 1 Institute of Evolutionary Biology, University of Edinburgh, Edinburgh, EH9 3FL UK; 2 Department for Evolutionary Genetics, Max Planck Institute for Evolutionary Biology, 24306 Plön, Germany

**Keywords:** Evolutionary genetics, Evolutionary biology, Inbreeding, Genetic variation, Mutation

## Abstract

For over a century, inbred mice have been used in many areas of genetics research to gain insight into the genetic variation underlying traits of interest. The generalizability of any genetic research study in inbred mice is dependent upon all individual mice being genetically identical, which in turn is dependent on the breeding designs of companies that supply inbred mice to researchers. Here, we compare whole-genome sequences from individuals of four commonly used inbred strains that were procured from either the colony nucleus or from a production colony (which can be as many as ten generations removed from the nucleus) of a large commercial breeder, in order to investigate the extent and nature of genetic variation within and between individuals. We found that individuals within strains are not isogenic, and there are differences in the levels of genetic variation that are explained by differences in the genetic distance from the colony nucleus. In addition, we employ a novel approach to mutation rate estimation based on the observed genetic variation and the expected site frequency spectrum at equilibrium, given a fully inbred breeding design. We find that it provides a reasonable per nucleotide mutation rate estimate when mice come from the colony nucleus (~7.9 × 10^−9^ in C3H/HeN), but substantially inflated estimates when mice come from production colonies.

## Introduction

Inbred mice have long been used for a wide variety of research purposes, ranging from biomedical to behavioral studies (Snell 1956; Boake 1994; Flint 2003; Lilue et al. 2019). Individuals of the same inbred strain are expected to be nearly genetically identical, and this is important for estimating the relative contribution of heredity and environment, the relative importance of different environmental factors on traits of interest, and for discovering the phenotypic effects of mutations (Russell 1941; Bailey 1982). Inbred strains allow experimenters to vary only the parameters of interest and to measure their effects (ruling out genetic variance), which is important for discovering causal factors and allowing experimental reproducibility (Beynen et al. 2001).

Mice have been inbred to reduce genetic variance for over a 100 years, and each generation of inbreeding is expected to lead to a decrease in heterozygosity (Wright 1921; Silver 1995). For example, in a breeding scheme beginning with two unrelated individuals (i.e., having no alleles shared identical by descent (IBD)), 50% of the individuals’ genomes are expected to be IBD after two generations of full-sib mating (Wright 1921, 1933; Green 1981). By 20 generations of full-sib mating, individuals are expected to be 99.8% IBD, and the strain is considered to be operationally inbred (Green 1981; Lyon and Searle Committee on Standardized Genetic Nomenclature of Mice Lyon and Searle 1989). Many important inbred strains used in laboratory studies today are lines that have been inbred by brother–sister mating for over 200 generations, by which time individuals within strains are expected to be homozygous at every site in the genome (Green 1966; Silver 1995). However, this expectation assumes that no spontaneous mutations arise, which would maintain genetic variation within lines, and that balancing selection does not maintain variation. Even in the absence of balancing selection, inbred lines are expected to maintain an equilibrium level of genetic variation (at mutation-drift balance), which is determined by the mutation rate and the effective population size (Watterson 1975).

When inbred mice are ordered from commercial breeders, they arrive from production colonies that are the endpoint of some standardized breeding scheme. These breeding schemes are designed to maintain the “genetic stability” of an inbred strain by reducing the effective number of generations (and, therefore, genetic differences) between mice purchased at different times (Flurkey and Currer 2009). The supplier of the mice used in this study (Janvier Labs, France) employs a pyramidal management scheme that is typical of the industry. Each strain is maintained as a cryopreserved colony nucleus, with the aim of slowing down the generation interval to one generation every 25–50 years instead of two generations per year. Embryos are periodically extracted from the colony nucleus to form an expansion colony, and from this, mice are transferred to a production colony, again expanding the number of individuals. Mice from the production colony are supplied to the end user, and this implies that there could be as many as ten generations between individuals (i.e., the number of generations before two individuals have common ancestors in the colony nucleus, going back in time). There will therefore be a buildup of genetic variation between individuals supplied from the production colony and the possibility of an increase in variation within individuals if strict full-sib mating is not adhered to either in the expansion or production colonies. This management scheme is similar to those used by other suppliers of inbred mice, such as the Jackson Laboratory, Envigo, Taconic Biosciences, and Charles River Laboratories.

It has long been known that there are genetic differences affecting traits among sublines of an inbred strain, but only recently, has this been investigated using whole-genome sequencing (Deol et al. 1960; Sittig et al. 2014; Dumont 2019). There is also evidence that mutations cause a change in many traits of interest within inbred lines (Åhlgren and Voikar 2019). A 20-generation, mutation accumulation study with whole-genome sequencing on a C57BL/6J subline of an inbred mouse, for example, found a 2.7% increase in the frequency of visible anomalies along with almost 300 new single nucleotide variants (Uchimura et al. 2015). The effect of the commercial breeding scheme on the isogenicity of individuals within strains has not been investigated, even though the genetic background of experimental mice can be important (Doetschman 2009; Casellas 2011; Leclercq and Kaminski 2015). Here, we sequence pairs of individuals of four commonly used inbred strains from different points in a commercial breeding scheme in order to quantify genetic variation within lines, and to test the isogenicity of these individuals across the genome. We then use the frequencies of variants observed within the lines to estimate mutation rates per nucleotide site per generation for these four strains, under the assumption that they are at mutation-drift balance.

## Methods

### Inbred strain acquisition and DNA extraction

One pair of mice of the C3H/HeN (C3H) strain was obtained from embryos directly from Janvier Labs’ colony nucleus, and one pair of mice of each of the three strains C57BL/6JRj (BL6), BALB/cAnNRj (BALBc), and FVB/NRj (FVB), were obtained from Janvier Labs’ production colonies (BL6 and BALBc as live mice and FVB as embryos). These mice from the production colonies are therefore expected to vary in their distance (in generations) from their colony nuclei. DNA was extracted from tail tissue of the eight inbred mice (one pair from each strain) using a standard salt extraction method that included an initial Proteinase K digestion step.

### Sequencing and alignment

Whole-genome sequencing was performed on the genomic DNA from the eight inbred mice using the Illumina SeqLab Platform at Edinburgh Genomics (Edinburgh, UK), which yielded >30× coverage for each individual (>120 Gb of 150-bp paired-end sequences). Reads were aligned to the *Mus musculus* reference genome (GRCm38) using BWA mem (0.7.13-r116). Data for each individual were then processed by the following bioinformatics pipeline: alignment sort using Samtools v1.9 (Li et al. 2009), synchronize read mate-pair information using Picard Tools v2.2, replace read groups using Picard Tools v2.2, mark duplicate reads using Picard Tools v2.2, and index using Samtools v1.9 (Li et al. 2009). This was followed by variant calling with HaplotypeCaller from GATK v4.1.2.0 (Poplin et al. 2018), and then the data from eight individuals, including data for nonvariant sites, were combined into one variant call format (VCF) file using CombineGVCFs and GenotypeGVCFs from GATK v4.1.2.0 (Poplin et al. 2018). Wild-mice whole-genome sequence data, obtained for 12 mice from a previous study (Harr et al. 2016), were used as “bait” for filtering variants, the idea being that if putative variants observed in the focal inbred mice are also present in the wild “bait” mice, then it is more likely that these “variants” are actually misaligned paralogous sequences than mutations in the focal inbred individuals. We have used a similar strategy previously (Ness et al. 2015). Bait mice included six *Mus musculus domesticus* individuals (four collected from France and two from Iran) and six *Mus musculus musculus* individuals (collected from Afghanistan). Their VCF files were reheaded to match the inbred line VCF files, and then CombineGVCFs and GenotypeGVCFs were used to create a combined wild-mouse VCF file using GATK v4.1.2.0 (Poplin et al. 2018).

### Variant identification

The objective here was to identify variant sites that were specific to one inbred strain only and also absent from the wild mice. We are therefore attempting to identify recent mutations segregating within individual inbred lines. Strain-specific variants were detected by filtering sites identified as having single nucleotide variants in one or both individuals of the same strain, while enforcing near purity in the inbred individuals of all other strains and all wild-mouse individuals. Each pair in a strain was subjected to this filtering in turn, when it was designated as the focal inbred pair. A site was considered to have a strain-specific variant if it met the following criteria:

1. Its PHRED-called site quality (QUAL) ≥ 90
2. The read depth of every inbred sample ≥10
3. The total number of variant reads in all wild-mice individuals ≤1
4. The total number of variant reads in all nonfocal inbred individuals ≤1
5. The genotype(s) of one or both sample(s) in only one inbred strain differed from the other inbred individuals, and there were ≤3 alternative alleles combined in the focal inbred pair
6. It is a single nucleotide variant

These criteria were coded into Python (v2.7.5) scripts, which incorporated the Cython wrapper cyvcf2 (Pedersen and Quinlan 2017). The strain-specific variant sites that passed the above criteria were then subjected to a manual check using the Integrative Genomics Viewer (IGV v2.5.0) with the following criteria:

(7) Heterozygous variant allele balance ratio ≥0.25
(8) Total read depth of variant site ≤2 × average genomic read depth
(9) Variants are not in phase with other variants
(10) It has no more than two alleles
(11) Variants do not have more than one read whose read pair was aligned to another chromosome.

Criteria 3 and 4 allowed for up to one read impurity among individuals (which were otherwise homozygous for the reference allele) due to possible sequencing errors. Haplotype-phase distance between variants in criterion 9 was determined by GATK v4.1.2.0 HaplotypeCaller “active site” defining algorithms (Poplin et al. 2018). Criteria 3 and 7 through 11 were specifically intended to filter out false positives due to misaligned paralogous reads, which were especially commonly observed where both individuals in the focal pair were heterozygous for the same variant. Many regions containing misaligned paralogous reads were recognizable because they tended to contain groups of linked variants in phase (determined by GATK v4.1.2.0 as criterion 9). Manual filtering criteria 7 through 11 were done on all C3H variants that passed the Python script filters, but only on a randomly sampled set of 100 passed variants in the cases of BALBc, BL6, and FVB (because it was not practical to manually filter the large number of variants detected). For the non-C3H strains, the total number of variants that would pass all criteria was estimated by multiplying the fraction of variants that passed manual filtering of 100 sampled by the number of variants that passed the automated Python script filters. Variant sites that satisfied criteria 1 through 6 only were considered pre-check sites, whereas variant sites that satisfied all 11 criteria were considered post-check sites. Mapping quality was not used as a filtering step because GATK HaplotypeCaller only provides the average mapping quality for variant sites (not for nonvariant sites) and, therefore, was not a filter that could be applied to both kinds of sites equally (but see Supplementary Table 1 for genome-wide averages).

### Expected variation simulations

In order to infer the expected level and pattern of variation at mutation-drift balance in an inbred line maintained by full-sib mating, and from this the mutation rate per site, simulations were run using SLiM3 (Haller and Messer 2019) to infer the unfolded site frequency spectrum (SFS). The SFS is a vector of counts of segregating sites of the possible genotypes in the two full sibs. Denoting the two full sibs sampled at mutation-drift balance as individuals A and B, the expected SFS at equilibrium is a vector **u** of seven elements, as defined in Table 1. Numbers of segregating sites in each of these seven categories were counted in the simulations. The total numbers of sites for each chromosome in the real data that passed the above filters (with the exception that they did not need to be variant) were used as the simulated chromosome sizes. Each simulation run started with an initially monomorphic population, which was then subject to 100 generations of neutral mutation with full-sib matings, at which point it was assumed that genotype frequencies were close to mutation-drift equilibrium. Population size was maintained at two individuals, the per-site mutation rate was set at 10^−8^ and the between-adjacent-site recombination rate was set at 5 × 10^−9^ (Cox et al. 2009). We also evaluated a range of other mutation- and recombination-rate parameter values, but these did not substantially change the inferred SFS proportions (Supplementary Figs. 1 and 2). The expected SFS was obtained from the simulations by averaging over 1000 replicates.

**Table 1:** Definition of the SFS.

SFS element	Definition of element
RARR	One alternative copy, individual A heterozygous
RRRA	One alternative copy, individual B heterozygous
RARA	Two alternative copies, both individuals A and B heterozygous
AARR	Two alternative copies, individual A homozygous alternative
RRAA	Two alternative copies, individual B homozygous alternative
AARA	Three alternative copies, individual B heterozygous
RAAA	Three alternative copies, individual A heterozygous

“R” and “A” under the SFS element refer to reference and alternative alleles, respectively.

### Mutation rate estimation

Estimates of the mutation rate for each inbred strain were obtained by comparing the observed SFSs for variant sites (**v**) with the expected SFS, **u**. For a given mutation rate in the experiment, *μ* (×10^−8^), the total squared difference between the observed SFS and scaled expected SFS elements was computed as follows:

$$d = {\Sigma}\left( {\mu u_i - v_i} \right)^2.$$

The golden section search algorithm was applied to find the value of *μ* that minimizes *d*. Ten iterations were sufficient to achieve convergence (to three significant digits). The 95% confidence intervals for the estimated mutation rates were obtained by bootstrapping one million times over the observed variants (sampling the same total number of segregating sites from SFS elements with replacement), and rerunning the golden section search for each resampling trial. This method was validated by comparing the observed number of variants for each strain with the number predicted by theory, assuming the estimated mutation rate for each strain (described in the next section).

### Expected number of variants per strain in an inbred line

To check that the mutation rate estimates based on the SFS predict the observed genetic diversity within each inbred strain, the estimated mutation rate for each strain was used to calculate the theoretical expectation for the amount of genetic diversity in an inbred line at mutation-drift equilibrium, i.e., 4*μN*_*e*_ × *L*, where *μ* is the mutation rate calculated above, *N*_*e*_ = 2.6, the effective population size of populations maintained by full-sib mating (Falconer 1960; Falconer and Mackay 1996), and *L* is the number of sites that passed quality filters (criteria 1 through 4 above) (Supplementary Table 2). This theoretical expectation for genetic diversity was then compared against the observed number of variant sites in each strain. We did not include other metrics that would require more than just the sample of 100 variants that were verified for each strain (BL6, BALBc, and FVB) since these were specifically filtered to exclude variants found in more than one strain. Therefore, the variants identified would show no overlap and would be completely differentiated in any population structure analysis.

## Results

### Observed and expected SFS per strain

**Fig. 1:**
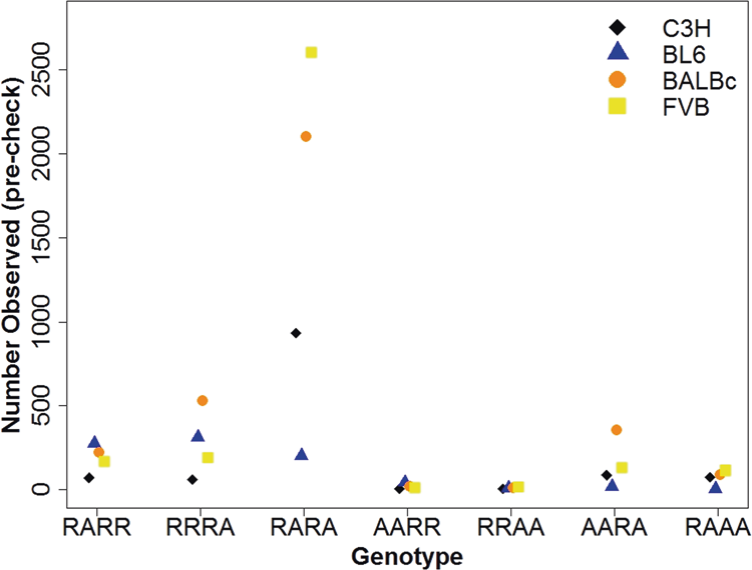
Observed number of variant sites for each SFS element pre-check. Observed numbers separated by inbred strain and taken after initial filtering but before manual IGV checks.

There was substantial variation in the number of single nucleotide variant sites observed among the mouse strains, especially in variants where both individuals in the same strain are heterozygous (RARA element in Table 1) (Fig. 1). BL6 (the strain on which the reference genome is based) has the lowest number of variant sites of this type, which is consistent with there being the smallest number of unmapped paralogs in this strain. In contrast, FVB has the highest number of RARA sites, more than 12 times the number observed in BL6. This is presumably because FVB is the most genetically distant from the reference genome of the four strains (Yang et al. 2011).

### Expected SFS from simulations

**Fig. 2:**
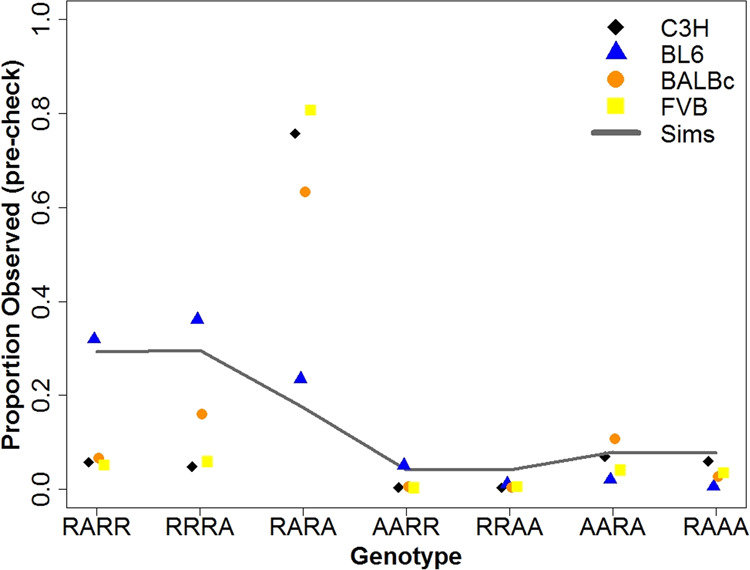
Proportion of variant sites for each SFS element in the four inbred strains observed after initial filtering but before manual IGV checks. Solid line: average proportion of variant sites in each SFS element after 100 generations in simulations with 2725,521,370 sites. SEMs for each element were all <2.2%.

In simulations, the relative values of the SFS elements were approximately constant by ~25 generations, so 100 generations were therefore adequate for the purpose of approaching equilibrium. The SFS elements with one alternative allele (RARR and RRRA) made up the majority of variants (Fig. 2). The double heterozygous genotype (RARA) had a similar frequency to the three alternative allele combined-genotype frequencies (AARA and RAAA), and was almost twice as frequent as that of the two alternative alleles in one individual genotype (AARR and RRAA).

### Comparison of the observed genotype frequencies to frequencies expected from simulations

When the observed genotype frequencies were compared with those expected in simulations, the double heterozygous genotype (RARA) was highly overrepresented in the observed data, except in the case of BL6 (Fig. 2). This is likely due to paralogous sequence misalignment, which tends to occur when a focal inbred strain has a duplicated region in its genome, but only one duplicate is present in the reference genome. In this case, reads from both duplicates align to only one region of the reference. If there are sequence differences between the duplicates, these appear as heterozygous sites. Some duplicates are strain-specific, so the problem is expected to increase with increasing distance from the reference genome. To reduce the problem of misaligned duplicated regions, we therefore did not include the RARA SFS element when estimating mutation rate.

### Number of variants observed per strain and per individual (post check)

**Fig. 3:**
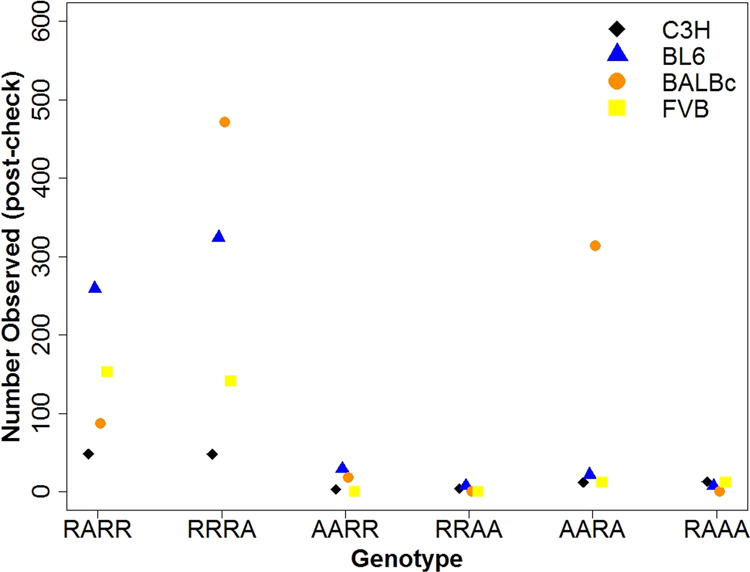
Observed number of variant sites for each SFS element post-check. Observed (for the C3H strain) and estimated numbers of sites (for the BL6, BALBc and FVB strains) in each SFS element (excluding the double heterozygote category, RARA) for each strain after initial filtering and manual IGV checks.

**Fig. 4:**
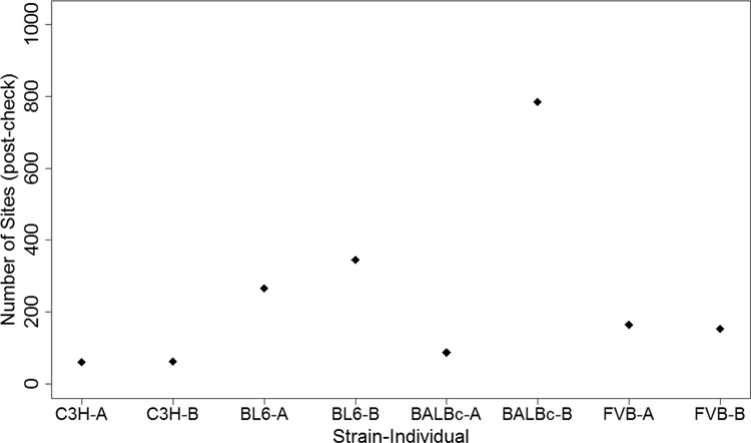
Observed number of variant sites per individual post-check. Observed (for the C3H strain) and estimated numbers of sites (for the BL6, BALBc and FVB strains) for each SFS element (excluding sites where both individuals in a strain are heterozygous, RARA) and for each individual after initial filtering and manual IGV checks.

After manual filtering, BL6 has a lower proportion of false positives (i.e., there were more sites that passed the filters) across the genome in sampled sites (98%) compared to BALBc and FVB, and the latter had the most false positives (73% and 52%, respectively) (Supplementary Fig. 3). After filtering, BALBc has the highest number of single nucleotide variant sites. Both BL6 and FVB have more variable sites than C3H, in cases where only one individual is heterozygous (RARR and RRRA). Surprisingly, BALBc has many more instances where individual B is heterozygous compared to individual A (compare the SFS element RARR with RRRA and element AARA with RAAA in Fig. 3). This pattern is also apparent in the total number of variant sites per individual (after manual filtering), i.e., BALBc individual B has about nine times the number of variants than individual A (Fig. 4). For the other three strains, there are only small differences in the number of variants between individuals of the same strain.

### Estimated mutation rates

**Fig. 5:**
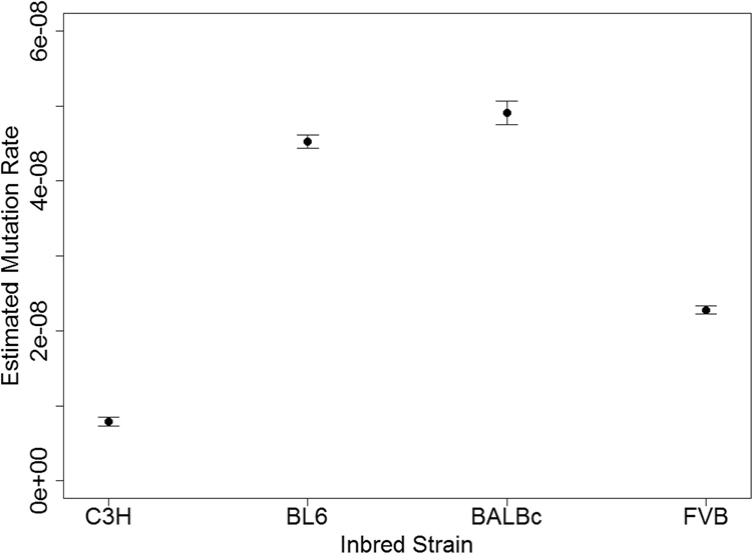
Estimated mutation rates based on the site frequency spectrum. Error bars indicate 95% confidence intervals by bootstrapping 1,000,000 times over SFS elements with the same total number of observed sites.

As described above, mutation rates were estimated for a given strain by minimizing the mean-squared difference between the observed SFS elements and those obtained by simulation. The simulations assume mutation-drift equilibrium, and that the SFS elements, expressed as proportions, are insensitive to the mutation rate assumed. BALBc and BL6 were estimated to have a mutation rate approximately six times higher than C3H (Fig. 5). FVB had a lower mutation rate, closer to three times that of C3H. The estimated mutation rate in C3H is ~7.9 × 10^−9^ (CIs: 7.33‒8.47 × 10^−9^).

### Expected amount of genetic variation

**Fig. 6:**
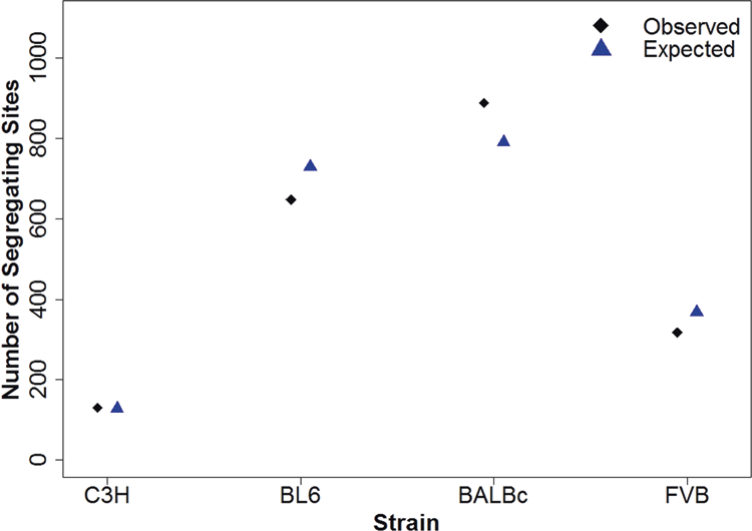
Comparison of observed and expected number of variant sites. Observed number of variant sites per strain (after initial filtering and manual IGV checks) and expected number of segregating sites obtained from estimated mutation rate and 4*μN*_*e*_ × *L*.

To check the effectiveness of estimating the mutation rate with the SFS, we used the estimated mutation rates to calculate the expected number of variants in each strain, and compared these to the observed numbers. The C3H strain had the closest number of observed variants to the expected number, whereas numbers in the other strains were either slightly over- or underestimated (Fig. 6). This was likely due to C3H individuals coming from the colony nucleus, where full-sib mating was more strictly adhered to.

## Discussion

The main difficulty in this analysis was the existence of a large excess (in comparison to the simulated proportions) of double heterozygous sites (RARA) after the initial alignment and genotyping in three of the four inbred strains. This excess was not observed, however, in the BL6 strain, on which the reference genome is based, so it is likely that we were actually observing false positives due to misaligned paralogous sequences (although there may be some expected differentiation from a reference genome that is two decades older than the samples) (Sarsani et al. 2019). Paralogous sequences are expected to be common, because the mouse genome is highly repetitive, consisting of >40% interspersed repetitive elements (Waterston et al. 2002; Komissarov et al. 2011; Bao et al. 2015). Repetitive sequences make alignment difficult, because sequences that are absent from the reference genome will tend to be misaligned to their paralogs. This potentially makes the two focal individuals of a given strain appear to be heterozygous, and explains why we observe such an excess of the double heterozygous SFS element, but not of the other SFS elements. Paralogous sequence misalignment appears to be exacerbated when there is a high level of sequence divergence from the reference genome. We see evidence of this when we compare the number of double heterozygotes observed in each strain with the previously reported phylogenetic distances from BL6 (which is closest to the reference). For example, FVB was reported to be genetically furthest from the reference and also had the highest number of double heterozygous sites in our study, whereas BALBc and C3H are more closely related to BL6 and had fewer double heterozygous sites (Beck et al. 2000; Zhang et al. 2005; Yang et al. 2011). It should also be noted that heterozygosity may be maintained by balancing selection, but this requires more than one breeding pair per generation and strong selection in order to be maintained in the face of genetic drift in inbred populations with very low effective population sizes (Robertson 1962; Bailey 1982). Although it is possible that some of the double heterozygotes we observed are the result of balancing selection, and this would have the effect of increasing mutation rate estimates, we do not expect it to be a major contributor to the estimated number of *de novo* mutations.

We attempted to filter false positives due to misaligned paralogous sequences using various strategies. The first was to use unrelated wild mice genomes as “bait” to filter sites that were variant in the focal pair and wild mice. This would remove many variants that had accumulated in repetitive regions, which are more likely to be misaligned (Ness et al. 2015). This bait-filtering step removed ~10% of sites that passed initial quality checks (when 12 baits from multiple origins and subspecies were used). The number of bait individuals (as well as the level of genetic differentiation between the bait chosen and the reference genome) will affect the number of sites that pass the bait filter, but as we increased the number of baits beyond 12, we saw diminishing returns on the number of sites filtered. It is certainly possible that this bait-filtering step might differentially remove false positives from the different strains, but we saw no evidence of this when we varied the subspecies and/or number of individuals used as bait. Another more general strategy was to filter sites by some maximum read depth. Here, we assumed that any site with read depth greater than twice the average genomic read depth was the result of reads misaligned to paralogous regions. This was the same reasoning as employed for filtering sites where more than two alleles were present: we should not expect to see this pattern within two individuals of the same strain, unless a paralogous sequence was misaligned. Two other filters, specifically designed to catch misaligned paralogs, were when variants were in phase with other variants, and when variants had reads whose read pair was aligned to another chromosome. Both of these were commonly associated with double heterozygous sites, and were assumed to be the result of paralogous misalignment. Overall, we believe that these filtering strategies were effective in substantially reducing false positives, because the number of post-check variants observed in BL6 was similar or higher than the observed numbers in the other strains (Fig. 4). In the future, it may be possible to obtain better alignments from hierarchical shotgun sequencing methods, but these are more laborious to perform, especially with many individuals (Waterston et al. 2002). This issue may also be alleviated as more complete genome references become available for strains other than C57BL/6J (Lilue et al. 2019). It should be noted that although we were aware that variants that matched patterns of misalignment (both individuals in the focal pair were heterozygous for the same variant that was often in groups and in phase with each other) were common enough to warrant methods to filter them out, we are not able to distinguish whether any specific variant is a true or false positive (beyond 100 sampled for BL6, BALBc, and FVB). Therefore, determining the number of true or false positives found in (non-)coding regions could only be grossly estimated using the overall proportions of coding and noncoding sites.

The C3H strain had the fewest number of segregating sites after all filters and checks were performed. This is presumably because the C3H individuals came directly from the colony nucleus, whereas individuals of the other strains came from production lines, which could vary in genetic distance from the nucleus (by up to ten generations, Flurkey and Currer 2009). The further a particular mouse ordered from a commercial breeder is from the nucleus, the higher the probability that mutations may have accumulated. This difference in distance from the nucleus presumably accounts for the approximately sixfold difference between the number of segregating sites observed in the C3H strain and in the other strains. We expect that a similar amount of variation may exist in inbred individuals from all commercial breeders, since they have similar strategies for maintaining inbred strains.

Perhaps more surprising was the observation that there was a large difference in the number of segregating variants within the BALBc strain, where one individual had approximately nine times the number of variants as the other. It is possible that the individual with many more variant sites came from generations further removed from the nucleus than the other individual. Alternatively, the difference could also be the result of non-full-sib mating that occurred somewhere during the expansion colony or production phase of commercial breeding. Although commercial breeders attempt to maintain brother–sister mating throughout their breeding schemes, pedigrees are not tracked in the production phase of breeding, so brother–sister mating cannot be guaranteed in the last five or so generations. As was observed in our study, if a sample of individuals are the result of mating between parents from even slightly diverged sublines, they may harbor more heterozygous sites. If we use the estimated mutation rate from the nucleus individuals (C3H) and the observed number of variants in the other strains, we can estimate their effective population sizes as N_e_ = *θ*/(4*μ* × *L*). These N_e_ values are 13, 18, and 6.5 for BL6, BALBc, and FVB, respectively. Although major breeders carry out regular quality controls of sample individuals within inbred strains to ensure genetic homogeneity, this is done with SNP panels that test a very small fraction of the genome (e.g., 27 SNPs at Jackson Labs, 32 SNPs at Charles River, 48 SNPs at Envigo, 96 SNPs at Taconic Biosciences, and 2050 SNPs at Janvier Labs). Even genome scans involving thousands of SNPs would not be enough. Whole-genome sequencing would be necessary in order to be certain that any two individuals within the same strain are genetically identical, something breeders are themselves transparent about, but which is not cost-effective.

We estimated the per nucleotide per-generation mutation rate in the C3H strain to be 7.9 × 10^−9^ (CIs: 7.3‒8.5 × 10^−9^) based on analysis of the SFS. We believe that the mutation rate estimate for the C3H strain is the only credible one in our study, because individuals of the other strains did not come from the colony nucleus, and therefore their estimates are inflated by *de novo* mutations that accrued during the expansion and production phases of the breeding design. For comparison, there are only a few other studies that have leveraged whole-genome sequencing to measure mutation rates in inbred strains of mice (Table 2). Lindsay et al. (2019) and Adewoye et al. (2015) sequenced several groups of inbred strain parents and their offspring and estimated mutation rates of 3.9 × 10^−9^ (CIs: 3.7‒4.2 × 10^−9^) and 3.8 × 10^−9^ (CIs: 3.0‒4.6 × 10^−9^), respectively, and Miholland et al. (2017) did a similar study with two parents and two offspring, resulting in an estimate between 6.7 and 7.0 × 10^−9^. In these three cases, per nucleotide per-generation mutation rates were calculated using the number of unique SNVs observed in the offspring and the number of nucleotides that passed filtering. In the latter study, the estimated mutation rate was deflated by removing 25% of the called mutations due to an expected false-discovery rate of 0.25. Another study was based on data from a mutation accumulation experiment that had been running for more than 20 generations, and used two different methods for estimating the per nucleotide per-generation mutation rate (Uchimura et al. 2015). One method used observed numbers of SNVs from the final generation of their experiment and an expected coalescence time for the SNV alleles in order to estimate the number of generations and then the per-generation mutation rate, which was 5.4 × 10^−9^ (CIs: 4.6‒6.5 × 10^−9^) per nucleotide. The other method sampled SNVs from the whole-genome sequencing data and performed targeted sequencing for the final generations, partly as a validation and partly as another estimation, and estimated a per nucleotide per-generation mutation rate of 6.8‒6.9 × 10^−9^. Eöry et al. (2010) employed a phylogenetic approach using synonymous sites in available sequences from both mice and rats to find a lower estimate in the range of 3.0‒3.2 × 10^−9^. This low estimate could be due to the synonymous substitution rate being lower than the mutation rate (i.e., because the neutral model is violated). Finally, an extreme upper mutation rate estimate of 3.7 × 10^−8^ was calculated by Lynch (2010) using target sequence data collected from various sources. It is unclear why their estimate is so high, but it may be due to underestimation of the number of generations involved. When compared to these previous results, our estimate of the C3H strain mutation rate is on the higher end of the estimates that used whole-genome sequences, but of the same magnitude. A high estimate could be the result of non-brother–sister mating somewhere during maintenance of the strain, but may also be somatic mutations that accumulated in the tissue of the sampled individuals in this study, which complicates comparisons between different tissues and ages (Blokzijl et al. 2016; Miholland et al. 2017). On the other hand, our similar estimation of magnitude to previous ones lends credence to our novel method of mutation rate estimation using the SFS (in combination with filtering false positives using wild “bait” mice), especially when information from previous generations is not available.

**Table 2:** Estimated per nucleotide per-generation mutation rates in mice from multiple studies using different estimation methods.

Samples	Sequence data	Estimated *μ* (×10^−9^)	Estimation method	Reference
C3H/HeNj (JAN)	Whole-genome sequencing	7.9 (CIs: 7.3‒8.5)	Site frequency spectrum	This study
C57BL/6J (CRL)	Whole-genome sequencing	5.4 (CIs: 4.6‒6.5)	Expected coalescence time	Uchimura et al. (2015)
C57BL/6J (CRL)	Sanger sequencing	6.8‒6.9	One generation	Uchimura et al. (2015)
C57BL/6 (NR)	Whole-genome sequencing	6.7‒7.0^a^	One-generation pedigree	Milholland et al. (2017)
CB57BL/6 × 129S5 (WSI)	Whole-genome sequencing	3.9 (CIs: 3.7‒4.2)	One-generation pedigree	Lindsay et al. (2019)
CB57BL/6 × CBA/Ca (HSD)	Whole-genome sequencing	3.8 (CIs: 3.0‒4.6)	One-generation pedigree	Adewoye et al. (2015)
Murids (UCSC)	Assembled genomes	3.0‒3.2	Phylogenetic	Eőry et al. (2010)
Mus	Various target sequences	37	Average over various studies	Lynch (2010)

*CIs* 95% confidence intervals (when provided), *CRL* Charles River Labs, *HSD* Harlan Sprague Dawley Inc., *JAN* Janvier Labs, *NR* not reported, *UCSC* University of California, Santa Cruz, *WSI* Wellcome Trust Sanger Institute.

^a^Milholland et al. (2017) mutation rate estimation is based on an expected false-discovery rate of 0.25.

## Conclusion

We estimated the mutation rate based on the genome sequences of pairs of individuals from four inbred mouse strains using the SFS of observed single nucleotide variants. Our mutation rate estimate for the C3H/HeN individuals in our study, which were obtained from the colony nucleus of a commercial breeding design, was similar to those found in previous whole-genome sequencing studies. The other strains, which were obtained from the production phase, had elevated levels of single nucleotide variation that were likely the result of the accumulation of mutations during the production phase and/or non-brother–sister mating. This led to inflated mutation rate estimations that are less credible. Unfortunately, the goal of using completely isogenic lines to remove genetic heterogeneity from experiments is at odds with the number of individuals of a particular strain that need to be produced by breeding companies in order to fill the demand for those experiments. Therefore, we suggest caution when interpreting the results of experiments using mice from production lines, where isogenicity is assumed, unless whole-genome sequences of those mice have been performed and the results taken into account. We expect that our novel method of mutation rate estimation will be applicable when strict, full-sibling inbreeding has been maintained for at least 20 generations (the actual number will vary, depending on initial variation and stochastic fixation/loss of allelic variants).

## Supplementary information

Supplemental Information

## Data Availability

The data for this study will be made available online through the NCBI Sequence Read Archive (SRA). Project Accession Number: PRJNA645870.
