# Supplementary material for: Inbred lab mice are not isogenic: genetic variation within inbred strains used to infer the mutation rate per nucleotide site
Source: Heredity (Edinb). 2020 Aug 31;126(1):107–16. doi: 10.1038/s41437-020-00361-1 (PMC7852876; doi:10.1038/s41437-020-00361-1)
Supplement: Supplementary file 1 — Supplemental Information [file 41437_2020_361_MOESM1_ESM.pdf]

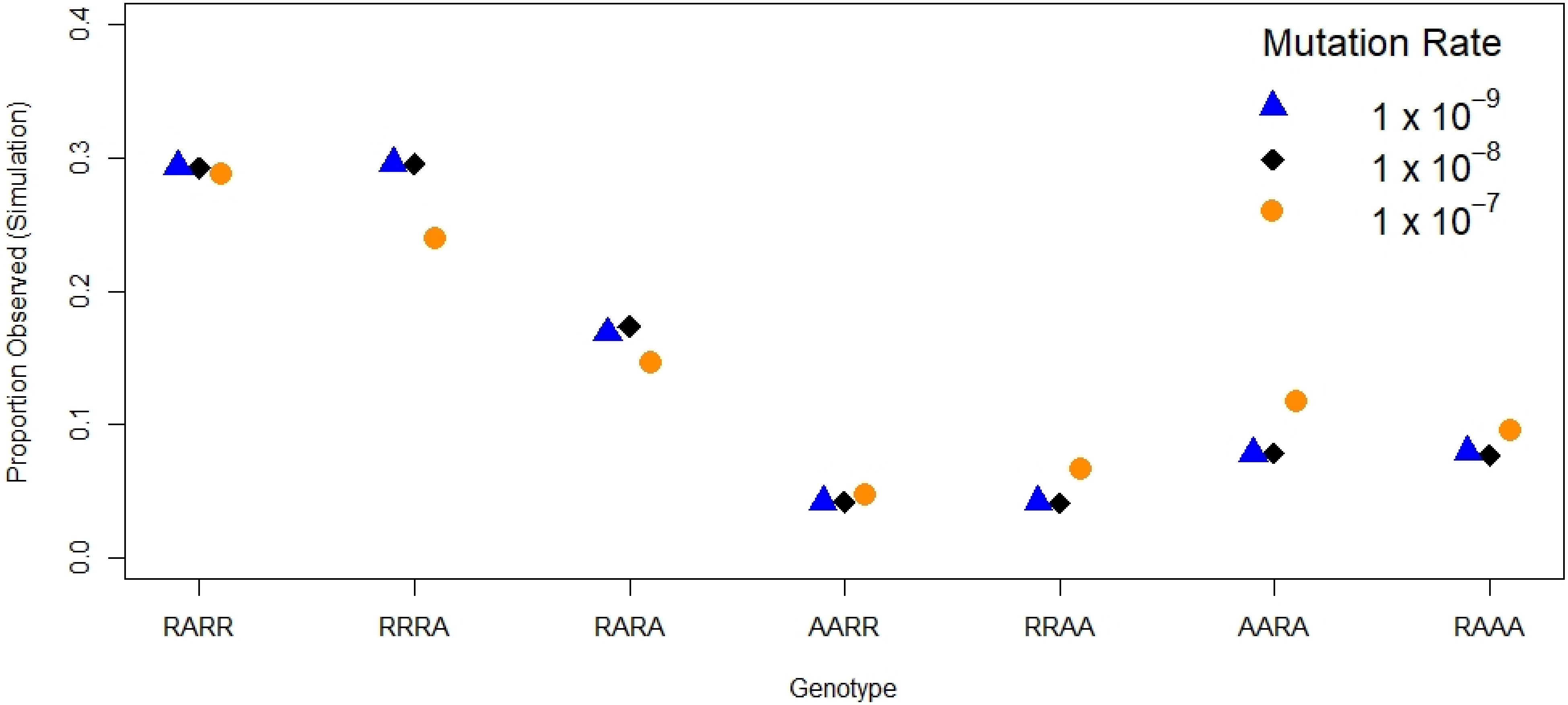

Supplemental Figure 1: Average proportion of variant sites in each SFS element after 100 generations in simulations in which mutation rate parameter values are varied. Parameter values include the value used in this study (10^{-8}), and values one order of magnitude smaller and greater.

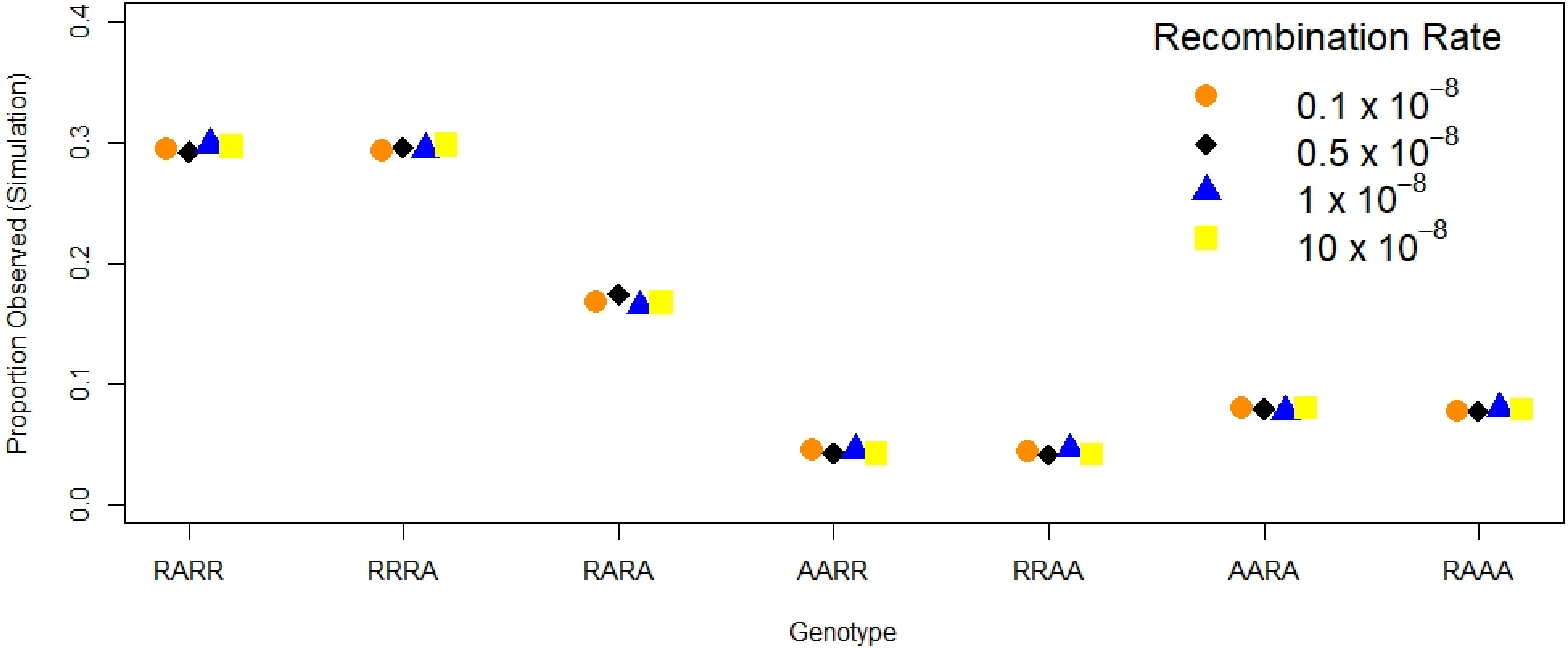

Supplemental Figure 2: Average proportion of variant sites in each SFS element after 100 generations in simulations in which recombination rate parameter values are varied. Parameter values include the value used in this study (0.5×10^{-8}) as well as lower and greater values.

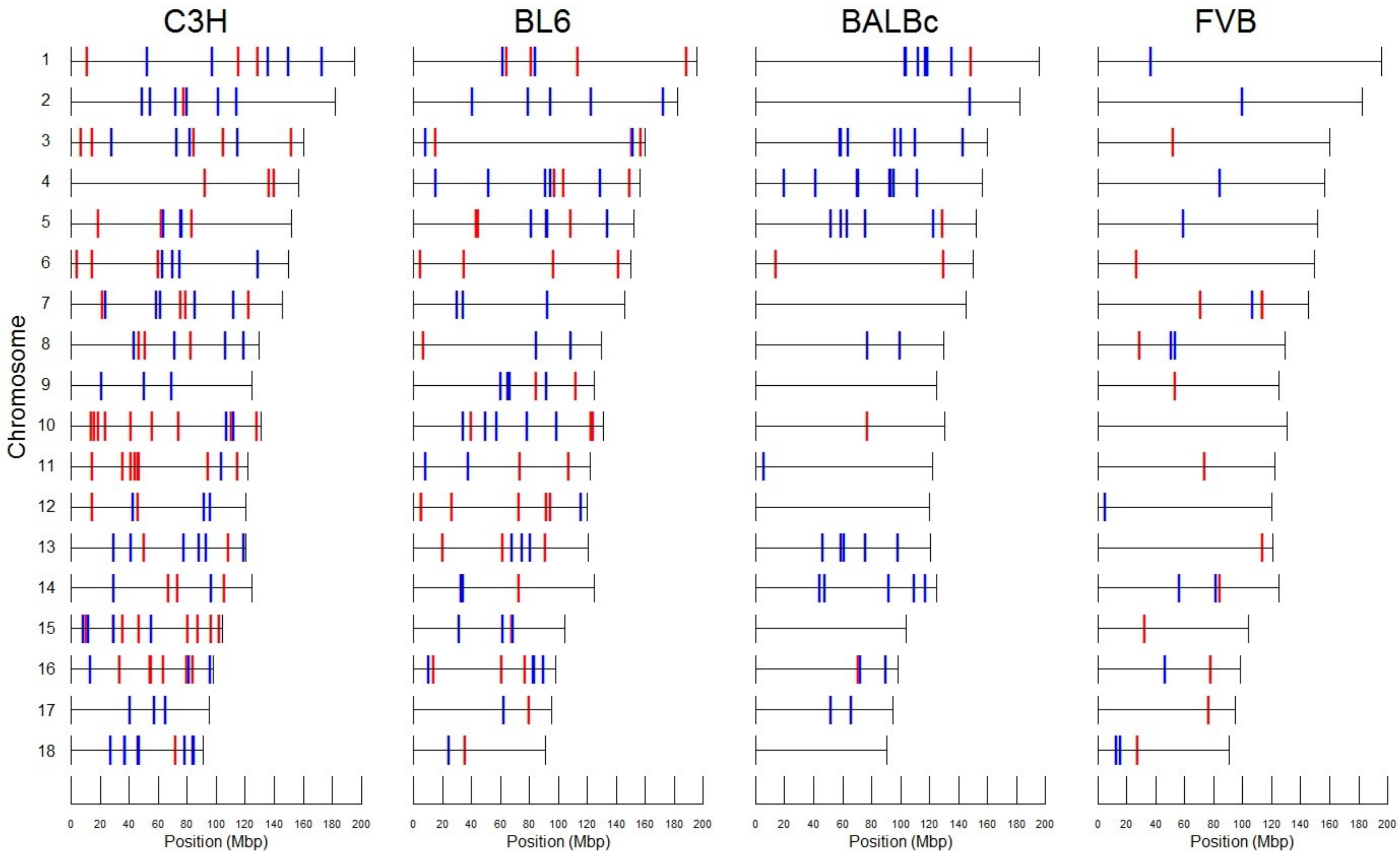

Supplemental Figure 3: Observed SNMs (post IGV check) across the genome in the four strains.

Supplemental Table 1. Mean sequence mapping quality for each sample.	
**Sample**	**Mean MAPQ**
C3H A	59.03
C3H B	59.06
BL6 A	58.69
BL6 B	58.64
BALBc A	58.94
BALBc B	58.91
FvB A	58.88
FvB B	58.66

Supplemental Table 2. Site counts during filtering process

Criterion	Filter	Number of Sites	% Initial
Initial	None	2,401,196,940	100
1 and 2	QUAL>90, DP>10	2,064,599,209	85.98
3	Bait mice < 1 variant reads	1,602,887,415	66.75
4	Non-focal mice < variant reads	1,543,509,803	64.28
5	Segregating sites in focal mice only	30,499	1.27E-03
6	Single nucleotide variant	8,648	3.60E-04
7 to 11	Manual filters for false positives on sample of sites (see methods)	1,984.45 (estimated)	8.26E-05
